# What about incorporating selenium in the therapeutic regimen of SARS-CoV-2?

**DOI:** 10.17179/excli2020-2968

**Published:** 2020-12-07

**Authors:** Bachir Benarba, Atanasio Pandiella

**Affiliations:** 1 Laboratory Research on Biological Systems and Geomatics, Faculty of Nature and Life Sciences, University of Mascara, Algeria; 2 Instituto de Biología Molecular y Celular del Cáncer, CSIC-IBSAL-Universidad de Salamanca, Campus Miguel de Unamuno, Salamanca, Spain

## ⁯

***Dear Editor,***

Since the COVID-19 outbreaks in China and due to the important number of infected cases and related deaths, various strategies have been proposed to be used to fight the pandemic including supplements (Krajewska et al., 2020[7]). In spite of several ongoing trials, no efficient drugs have been validated till now. An optimal and equilibrated immune response in infected individuals seems to be a promising preventive and therapeutic alternative. Actually, it has been suggested that besides the close relationship of Chinese populations with wildlife and related dietary traditions, selenium deficiency was the cause of the SARS-CoV-1 spread due to its involvement in viral mutations (Qureshi, 2016[12]). Selenium deficiency was suspected to be associated with enhanced virulence of SARS since previous studies have shown that this deficiency was linked to higher mutation rate and virulence of coxsackievirus B3 and influenza and poliovirus. Several studies showed that selenium supplementation resulted in enhanced immune response in adults with or without viral infections (Jayawardena et al., 2020[6]). Furthermore, such supplementation was found to effectively suppress the HIV-1 viral load (Hurwitz et al., 2007[4]). Indeed, selenium possesses an important antioxidant potential through its incorporation in various selenoenzymes such as glutathione peroxidase able to breakdown hydrogen peroxide (Medeiros-Neto and Rubio, 2016[9]). Accordingly, selenium deficiency, which is common among patients with viral infections, results in an enhanced reactive oxygen species production leading to an increased oxidative stress which promotes a decreased immune response, promotes viral mutations and an increased virulence. Selenium supplementation significantly improves the immunocompetence of infected hosts and decreases viral mutations leading to lowered virulence (Harthill, 2011[2]). These properties of selenium led to its incorporation into a novel patented antiviral treatment called Gene-Eden-VIR/Novirin (Polansky and Lori, 2020[11]). Furthermore, the immune boosting properties of selenium led to its incorporation into a herbal immuno-modulator drug used efficiently against viral respiratory diseases (Arastoo et al., 2014[1]). Interestingly, it has been recently demonstrated that selenium intake was positively associated with telomere length in adults (Shu et al., 2020[13]). It would also be interesting to experimentally analyze the action of selenium on viral infectiveness using *in vitro *and* in* vivo animal models.

Recently, selenium deficiency besides vitamin D deficiency was found to be characteristic of Korean COVID-19 patients. In fact, selenium deficiency was found in 42 of COVID-19 patients whereas their vitamins B, folate and zinc levels were not decreased. Interestingly, selenium deficiency was found in 100 of severe COVID-19 patients against 44.4 in those with mild COVID-19 (without pneumonia) suggesting that selenium deficiency may weaken the immune system and thereby result in increasing the severity of the disease (Im et al., 2020[5]). Likewise, Majeed et al. (2020[8]) found that Indian COVID-19 patients had lower selenium levels (69.2 ± 8.7 ng/ml) when compared to healthy individuals (79.1 ± 10.9 ng/ml). In another cross sectional study, Se (selenium) deficiency was found in 44.4 of COVID-19 German patients. On the other hand, Se deficiency was also found to be correlated with survival rates since 64.7 of non-survivors were deficient in Se against 39.96 of survivors (Moghaddam et al., 2020[10]). Furthermore, serum selenium levels were demonstrated to be an important predictor of survival among COVID-19 patients. Actually, it has been demonstrated that unimpaired Se transporter selenoprotein P and Zn status was predictor of high survival in COVID-19 patients suggesting that Se and/or Zn supplementation may be a promising approach (Heller et al., 2020[3]). Therefore, intervention studies are needed to investigate whether a Se supplementation may prevent severe complications of the COVID-19, and related death. Besides, the prognostic value of Se status using serum Se and SELENOP levels may be a promising alternative, especially in Se-deficient patients. 

Hence, we suggest that patients with COVID-19 may be at higher risk for selenium deficiency and, therefore, their selenium status should be assessed. Moreover, we suggest incorporating selenium supplementation to enhance their immune response and reduce the SARS-CoV-2 virulence. 

## Conflict of interest

The authors declare no conflict of interest.

## References

[1] Arastoo M, Khorshid H, Radmanesh R, Gharibdoust F (2014). Combination of IMOD™ and Arbidol to increase their immunomodulatory effects as a novel medicine to prevent and cure influenza and some other infectious diseases. J Med Hypothes.

[2] Harthill M (2011). Review: Micronutrient selenium deficiency influences evolution of some viral infectious diseases. Biol Trace Elem Res.

[3] Heller RA, Sun Q, Hackler J, Seelig J, Seibert L, Cherkezov A. (2020). Prediction of survival odds in COVID-19 by zinc, age and selenoprotein P as composite biomarker. Redox Biol.

[4] Hurwitz BE, Klaus JR, Llabre MM, Gonzalez A, Lawrence P, Maher KJ (2007). Suppression of human immunodeficiency virus type 1 viral load with selenium supplementation: a randomized controlled trial. Arch Intern Med.

[5] Im JH, Je YS, Baek J, Chung MH, Kwon HY, Lee JS (2020). Nutritional status of patients with COVID-19. Int J Infect Dis.

[6] Jayawardena R, Sooriyaarachchi P, Chourdakis M, Jeewandara C, Ranasinghe P (2020). Enhancing immunity in viral infections, with special emphasis on COVID-19: A review. Diabetes Metab Syndr.

[7] Krajewska J, Krajewski W, Zub K, Zatoński T (2020). COVID‑19 in otolaryngologist practice: A review of current knowledge. Eur Arch Otorhinolaryngol.

[8] Majeed M, Nagabhushanam K, Gowda S, Mundkur L (2020). An exploratory study of selenium status in normal subjects and COVID-19 patients in South Indian population: Case for adequate selenium status. Nutrition.

[9] Medeiros-Neto G, Rubio IGS, Jameson JL, De Groot LJ, de Kretser DM, et al. (2016). Iodine-deficiency disorders. Endocrinology: Adult and pediatric.

[10] Moghaddam A, Heller RA, Sun Q, Seelig J, Cherkezov A, Seibert L (2020). Selenium deficiency is associated with mortality risk from COVID-19. Nutrients.

[11] Polansky H, Lori G (2020). Coronavirus disease 2019 (COVID-19): First indication of efficacy of Gene-Eden-VIR/Novirin in SARS-CoV-2 infection. Int J Antimicrob Agents.

[12] Qureshi AI (2016). Ebola virus disease epidemic in light of other epidemics. Ebola Vir Dis.

[13] Shu Y, Wu M, Yang S, Wang Y, Li H (2020). Association of dietary selenium intake with telomere length in middle-aged and older adults. Clin Nutr.

